# Supplementary material for: Towards ultra-sensitive and rapid near-source wastewater-based epidemiology
Source: Nat Commun. 2025 Sep 1;16:8158. doi: 10.1038/s41467-025-63192-w (PMC12402315; doi:10.1038/s41467-025-63192-w)
Supplement: Supplementary file 1 — Supplementary Information [file 41467_2025_63192_MOESM1_ESM.pdf]

Supplementary Information

Title:
Ultra-sensitive and Rapid Near Source Wastewater-based Epidemiology

Authors:
Da Huang[1*], Alyssa Thomas DeCruz[1,2], Dounia Cherkaoui[1,2], Benjamin Miller[1*], Diluka Peiris[1], Samuel Hopgood[1,2], Jessica Kevill[3], Kata Farkas[3], Rachel Williams[3], Davey L. Jones[3], and Rachel A. McKendry[1,2*]

Affiliations:
1. London Centre for Nanotechnology, University College London, London WC1H 0AH, UK.
2. Division of Medicine, University College London, London WC1E 6BT, UK.
3. School of Environmental & Natural Sciences, Environment Centre Wales, Bangor University, Bangor, Gwynedd, LL57 2UW, UK.

Corresponding Author: *
Lead Corresponding Author:
Rachel A. McKendry | r.a.mckendry@ucl.ac.uk
Joint Corresponding Author:
Da Huang | d.huang@ucl.ac.uk
Benjamin Miller | ben.miller@ucl.ac.uk

Supplementary Table 1 A list of oligonucleotides used for RPA assay.

	Target	Name	Sequence (5'-3')
CNP Assay	E gene	Forward_E	[DIG]TTACACTAGCCATCCTTACTGCGCTTCGAT
		Reverse_E	[Biotin]GCTAAAATTAAAGTTCCAAACAGAAAAACT
	RdRp gene	Forward_RdRp	[FAM]GAGTGTGCTCAAGTATTGAGTGAAATGGTC
		Reverse_RdRp	[Biotin]CTGTGTTGTAAATTGCGGACATACTTATCG
FND Assay	E gene	Forward_E	[DIG]TTACACTAGCCATCCTTACTGCGCTTCGAT
		Reverse_E	[Biotin]GCTAAAATTAAAGTTCCAAACAGAAAAACT

Supplementary Table 2 A summary of the estimated costs of our prototype assay, and the comparison to the conventional assays. The listed costs for our dipstick assays are for prototyping and small orders. The Axxin reader was a bespoke version from the company with higher price than the market version. Many of these costs could be dramatically reduced by the economies of scale of mass-manufacturing.

	**Dipstick**			**PCR**		
	Carbon Nano Particles	FND – Axxin bespoke portable reader	FND – Smartphone reader (Miller *et al., Nature* 2020)	qPCR	ddPCR	Mobile PCR
Reagents cost (£, per test)	8.2	7.4	7.4	20-120	100-140	20-120
Instrument cost (£, reusable)	0	£6,700	£400 + Smartphone	£25,000 - £50,000	£65,000-£150,000	£10,000 £20,000

Note: all costs were estimated in Sterling Pounds (£). PCR cost estimates are based on a combination of publicly available information and direct quotations received from UK suppliers.

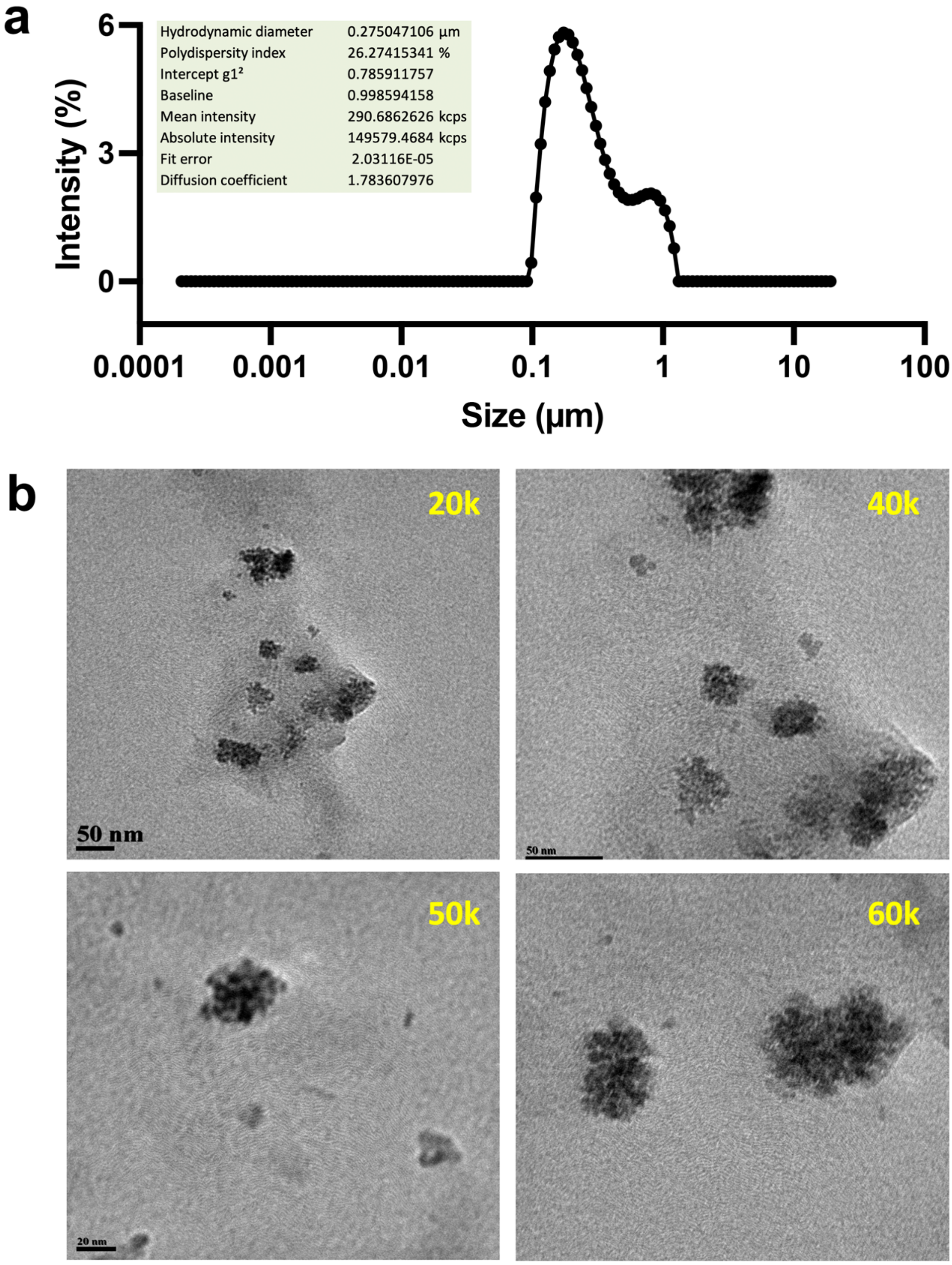

Supplementary Fig. 1 Characterisation of carbon nanoparticles. a Dynamic light scattering (DLS) analysis of carbon nanoparticles. The size distribution of the nanoparticles was ranging from 100 nm to 1.2 µm, indicating an uneven sizes of clusters formation. This is likely due to the particles rehydrating from the lyophilized conjugation pad. b Scanning electron microscopy (SEM) analysis of carbon nanoparticles. It was shown nanoparticles with uneven sizes across 20 nm to 50 nm. The clusters of particles was also revealed.

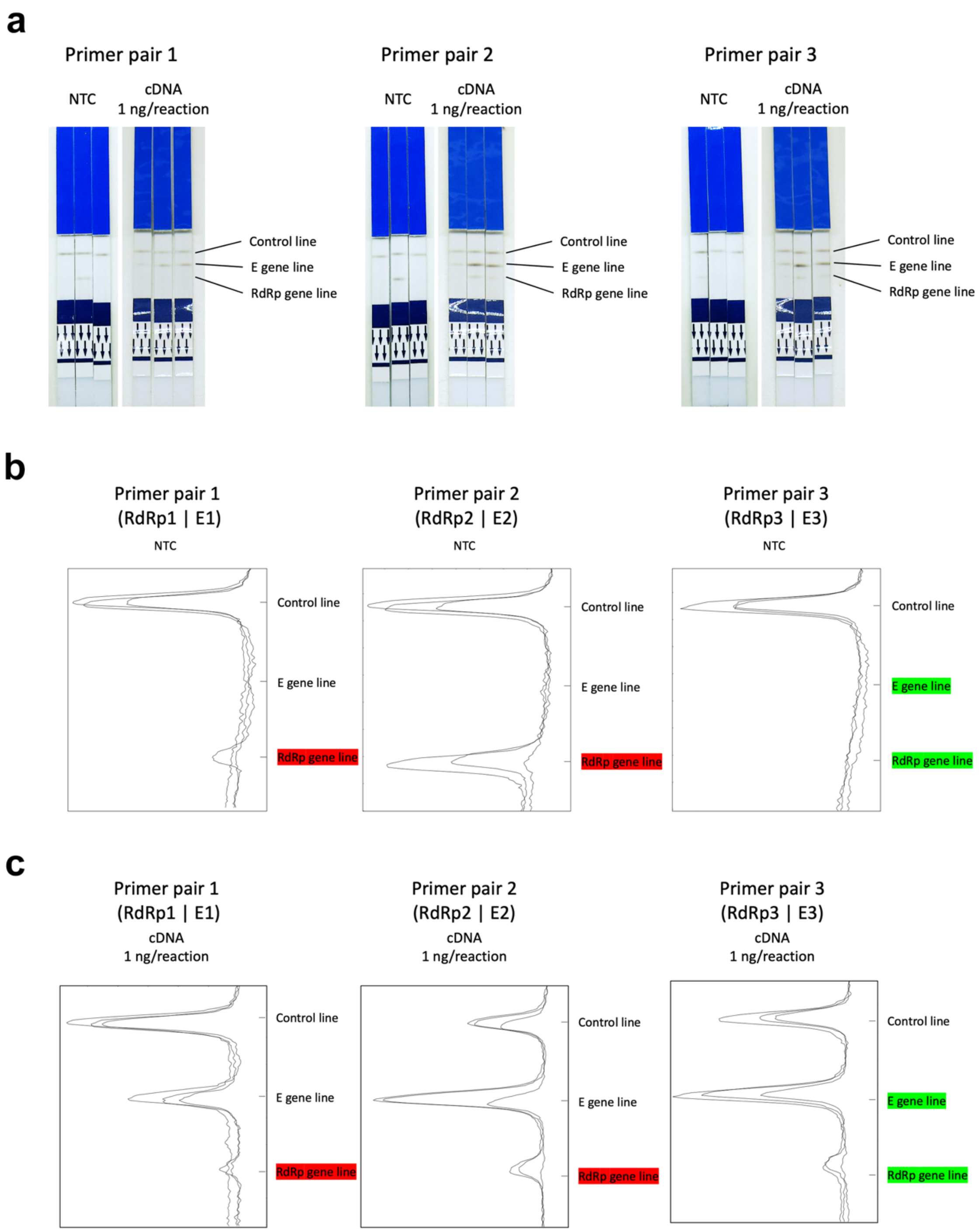

Supplementary Fig. 2 Screening of best primer pair via carbon nanoparticles dipstick. a Dipsticks captured images for NTC and cDNA samples, both E gene and RdRp gene were tested. b Test lines analysis of NTC samples: false positives were spotted on RdRp primer pairs. c Test lines analysis of cDNA samples: RdRp gene primers performed weaker than E gene primers. (n=3).

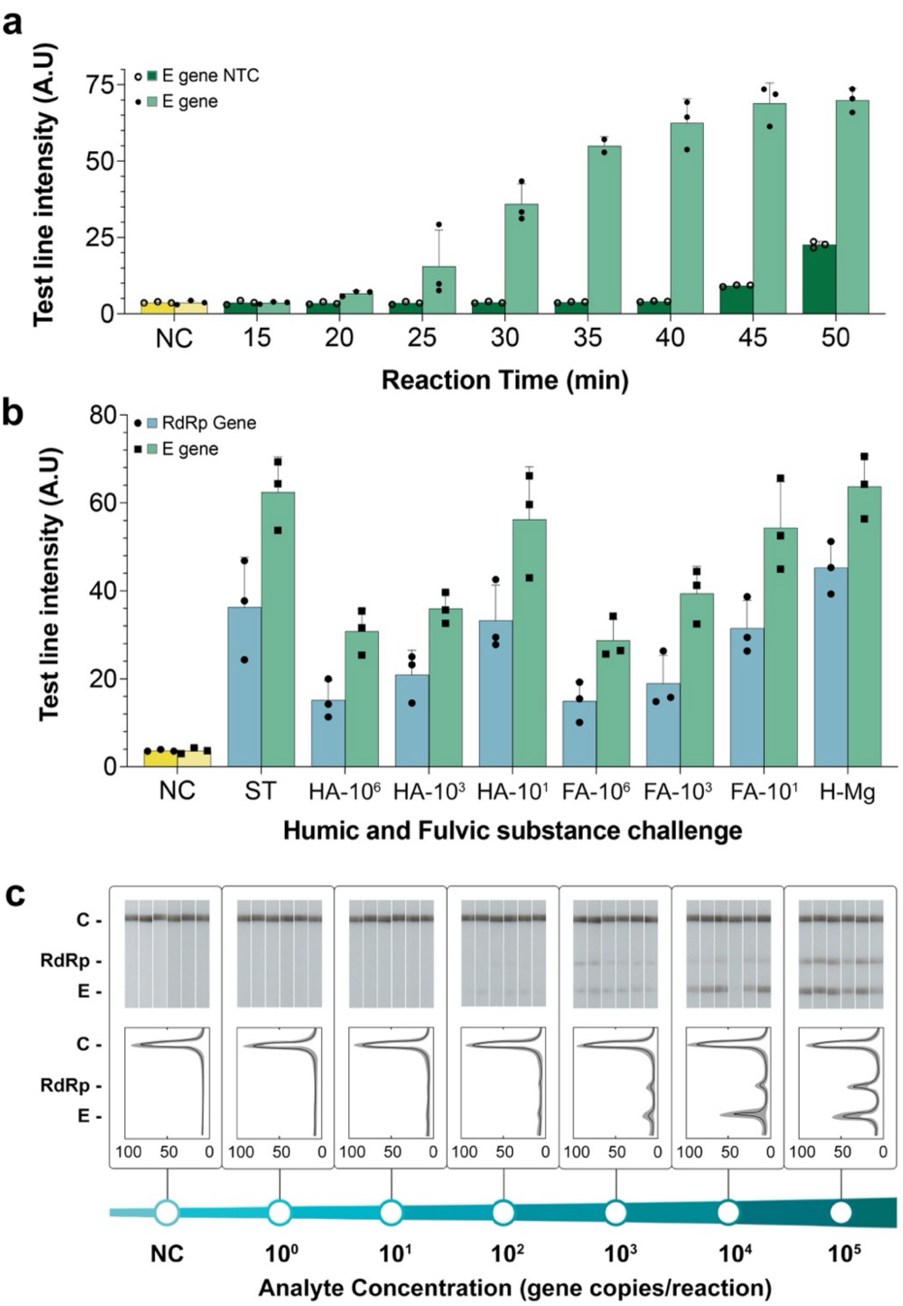

Supplementary Fig. 3 Development and optimisation of carbon nanoparticle assisted lateral flow assay. a Optimization of the RPA time-to-result. The signal increase with incubation time. After 40 min, reaction present the non-specific amplification. (n=3 technical replicates). **b** RPA assay resistance of Humic and Fulvic substance contaminants for the application n on wastewater. RPA assay represents the resistance to the chelation substance with minor moderation on low concentration of contaminants. Increase of the Mg^{2+} input in the reaction ease the deduction from the substance. The dots show means and error bars show the s.d. of repeat measurements (n=3 technical replicates). **c** Test strips capture images and analysis for measuring limits of detection for RPA-LFT-CNP assay for E gene and RdRp gene. Dilution series of amplicons were run on LFTs and imaged by the camera. The intensity of test lines were analysed in Matlab, and plotted, fitted to the Langmuir adsorption model. (n=3)

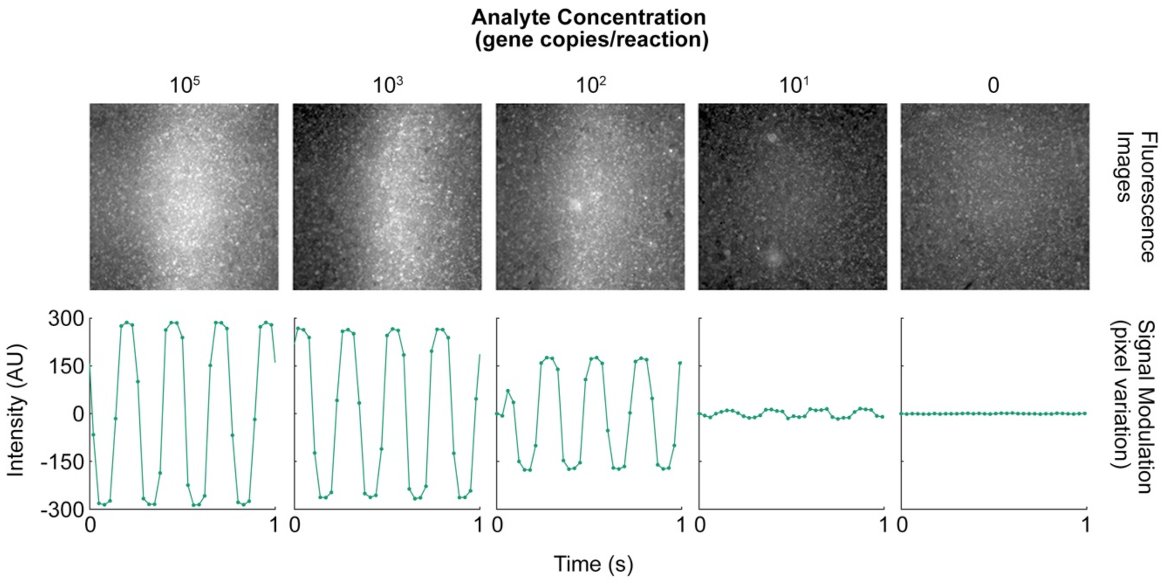

Supplementary Fig. 4 Extended comparison between lock-in and conventional fluorescence images analysis. This represents an example of nanodiamond fluorescence lock-in data acquisition from a series of image captures (see Method: Fluorescence measurements and analysis). On the top, it is the fluorescence images captured from lateral flow test strips, throughout series dilution of analyte concentration. On the bottom, it is the intensity–time plots represent signal modulation (pixel variation), showing that a periodic signal is still evident after the test line is no longer visible in the fluorescence images.

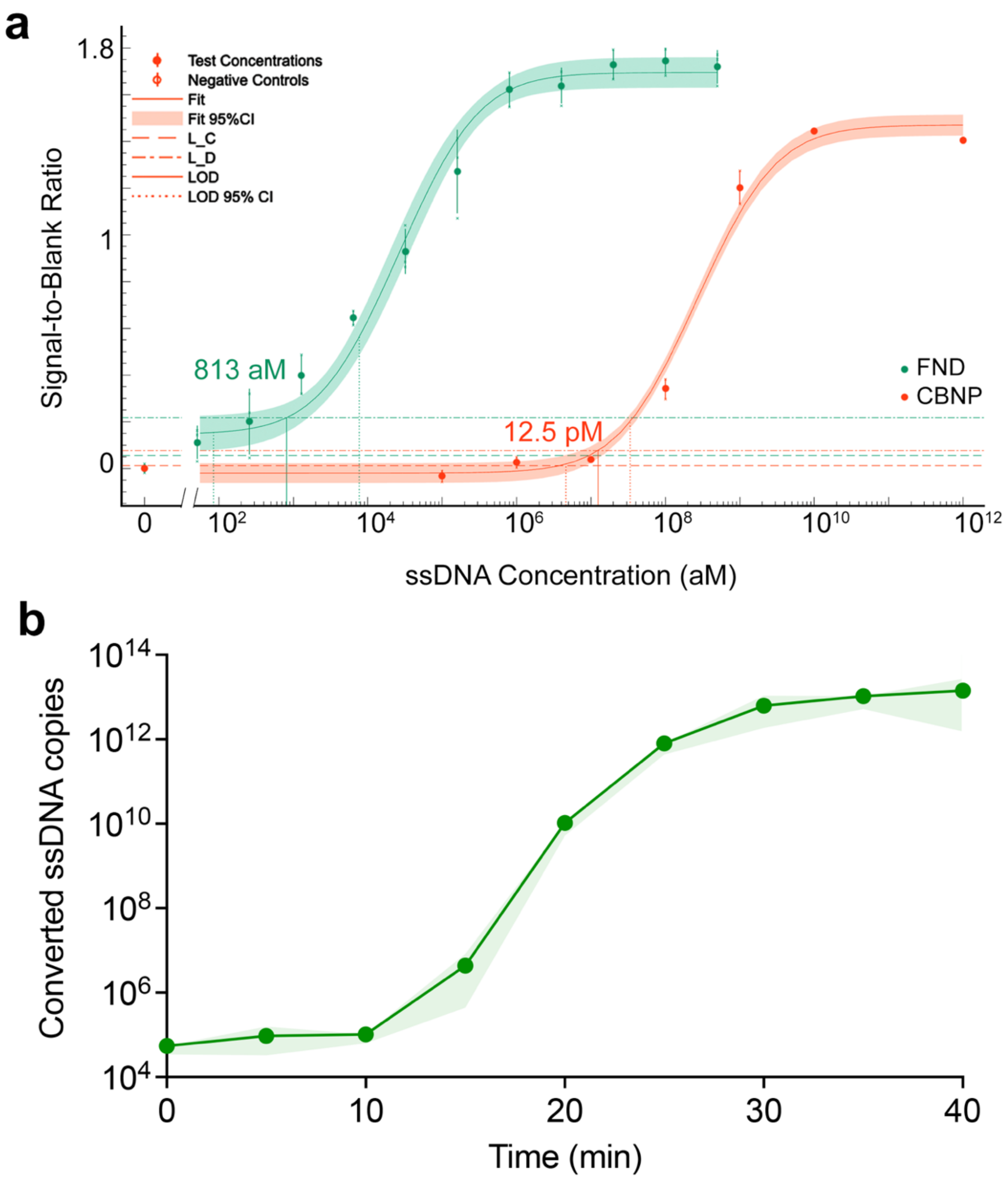

Supplementary Fig. 5 Development and optimisation of nanodiamond-enhanced lateral flow assay. a LoD comparison between FND and CBNP based on ssDNA model amplicon on LFT. The assay readout sensitivity improvement of 15375-fold over CBNPs to FNDs. The dots show means and error bars show the s.d. of repeat measurements (n=3 technical replicates and n=3 measurement replicates for each sample). A stretched exponential regressions are shown by solid lines, and shaded areas show the 95% confidence intervals of the fits. **b** Evaluation of converted ssDNA copy numbers over the RPA amplification based on the high concentration of target strands. This indicates an around 10000 folds changes in DNA copies over RPA in 20 minutes. As a result, 25 minutes of RPA amplification time can be applied for FND assay. The dots show means (n=3) and shaded areas show the ranging of the replicates.

a

CNP

Amplification 40min / CT: 25-40						
		RT-PCR Positive	RT-PCR Negative		Sensitivity (95% CI)	Specificity (95% CI)
RT-RPA	Positive	33	0	N		
	Negative	8	21	62	80% (65-91)	100% (84-100)

GNP

Amplification 40min /CT: 25-40						
		RT-PCR Positive	RT-PCR Negative		Sensitivity (95% CI)	Specificity (95% CI)
RT-RPA	Positive	27	0	N		
	Negative	6	18	51	82% (65-93)	100% (81-100)

FND Portable Reader

Amplification 25min / CT: 25-40						
		RT-PCR Positive	RT-PCR Negative		Sensitivity (95% CI)	Specificity (95% CI)
RT-RPA	Positive	39	0	N		
	Negative	2	21	62	95% (83-99)	100% (84-100)

FND Microscope

Amplification 25min / CT: 25-40						
		RT-PCR Positive	RT-PCR Negative		Sensitivity (95% CI)	Specificity (95% CI)
RT-RPA	Positive	41	0	N		
	Negative	0	21	62	100% (91-100)	100% (84-100)

b

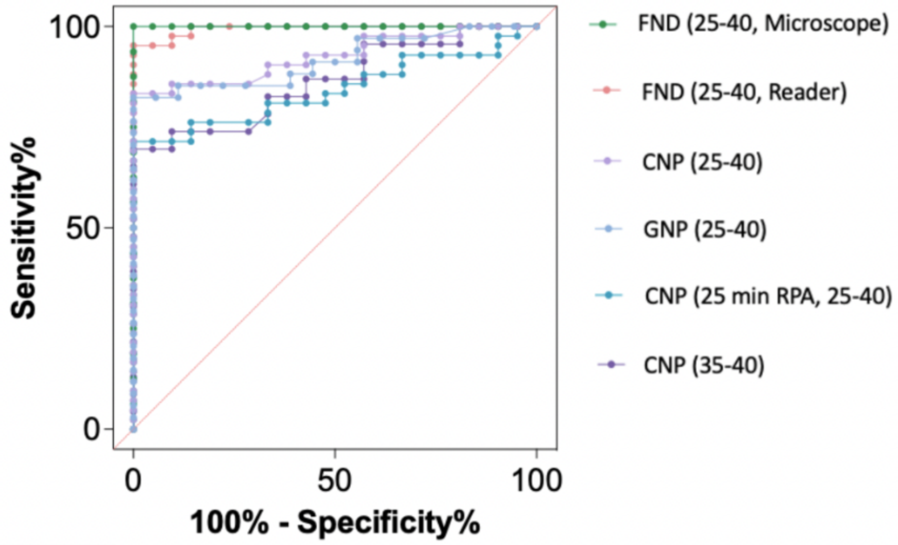

Supplementary Fig. 6 Analysis of LFT performances on different nanoparticles and readouts. **a** Sensitivity and specificity analysis of the different sensing conditions, including gold nanoparticles (GNP), carbon nanoparticles (CNP) and fluorescent nanodiamond (FND) assisted LFT, and FND test via portable reader readout and microscope readout. **b** ROC curve of the different sensing conditions. FND based LFT represents the highest sensitivity.

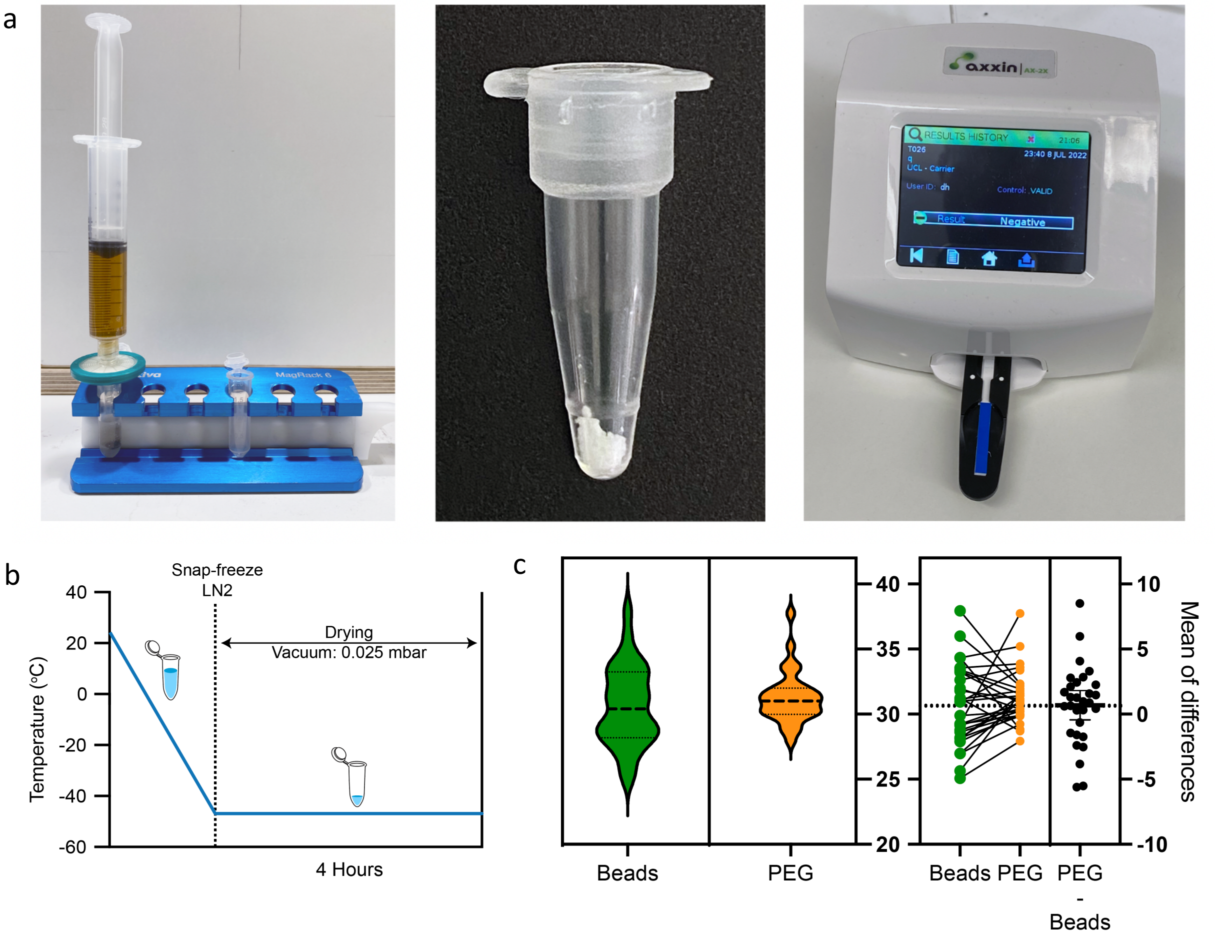

Supplementary Fig. 7 Development of lab in a suitcase. a Images of near-source testing setup. From left to right: syringe filter concentration with magnetic beads extraction (on magnetic rack), freeze-dried RPA reaction, and portable reader for FND-LFT strips. **b** Lyophilization of RPA reactions enhance the thermal stability of assay and reduce the steps of practise, allowing the near source testing. **c** Comparative analysis of RNA extraction efficiency between magnetic bead extraction and PEG precipitation methods. The graph shows qPCR C_t values for 30 paired wastewater samples processed using identical primers and methods (left). Statistical analysis using a paired two-tailed *t*-test revealed no significant difference in recovery efficiency between the two methods (P=0.2254, t=1.239) (right). While the magnetic bead method shows more variable recovery rates, it offers significant advantages in processing time, workflow simplicity, and field applicability, which is suitable for near-source testing applications.

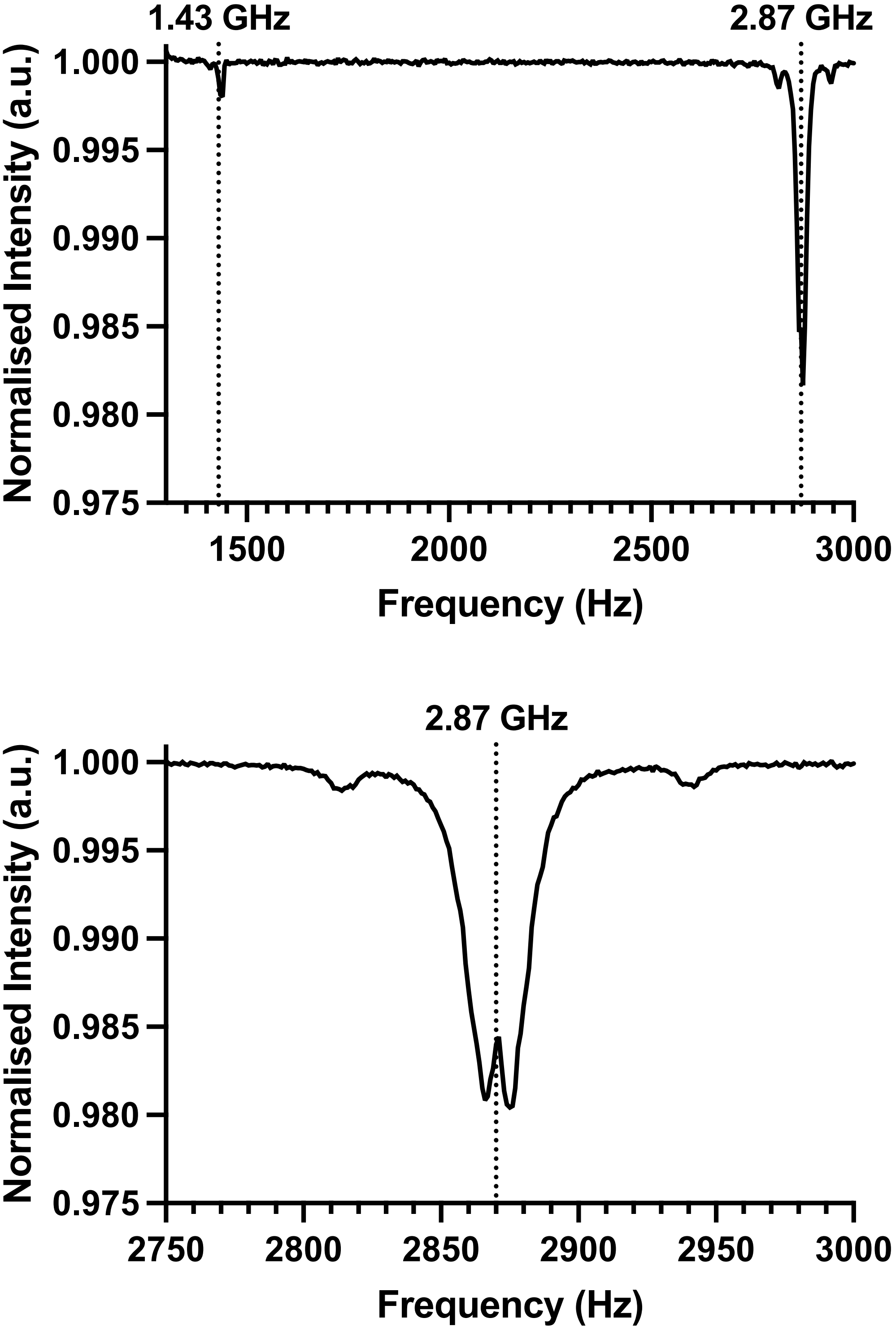

Supplementary Fig. 8 Continuous wave optically detected magnetic resonance (CW-ODMR) measurement of the nitrogen-vacancy (NV) centres in nanodiamond. (Top) The fluorescence signal decreases at approximately 2.87 GHz and 1.43 GHz, corresponding to the ground-state (ΔE= 2.87GHz) and optically excited-state (ΔE*= 1.43GHz) zero-field splittings, respectively. The fluorescence reduction is approximately 0.20% at 1.43 GHz and 1.8% at 2.87 GHz. (Bottom) Zoomed-in spectrum around 2.87 GHz, revealing the detailed structure of the ODMR dip.
