# Supplementary material for: Towards ultra-sensitive and rapid near-source wastewater-based epidemiology
Source: Nat Commun. 2025 Sep 1;16:8158. doi: 10.1038/s41467-025-63192-w (PMC12402315; doi:10.1038/s41467-025-63192-w)
Supplement: Supplementary file 2 — Reporting Summary [file 41467_2025_63192_MOESM2_ESM.pdf]

nature portfolio

Corresponding author(s): DAPR NCOMMS-24-82604A

Last updated by author(s): Aug 11, 2025

Reporting Summary

Nature Portfolio wishes to improve the reproducibility of the work that we publish. This form provides structure for consistency and transparency in reporting. For further information on Nature Portfolio policies, see our Editorial Policies and the Editorial Policy Checklist.

Statistics

For all statistical analyses, confirm that the following items are present in the figure legend, table legend, main text, or Methods section.

n/a	Confirmed	
☐	☒	The exact sample size (*n*) for each experimental group/condition, given as a discrete number and unit of measurement
☐	☒	A statement on whether measurements were taken from distinct samples or whether the same sample was measured repeatedly
☐	☒	The statistical test(s) used AND whether they are one- or two-sided<br>*Only common tests should be described solely by name; describe more complex techniques in the Methods section.*
☒	☐	A description of all covariates tested
☐	☒	A description of any assumptions or corrections, such as tests of normality and adjustment for multiple comparisons
☐	☒	A full description of the statistical parameters including central tendency (e.g. means) or other basic estimates (e.g. regression coefficient) AND variation (e.g. standard deviation) or associated estimates of uncertainty (e.g. confidence intervals)
☐	☒	For null hypothesis testing, the test statistic (e.g. *F*, *t*, *r*) with confidence intervals, effect sizes, degrees of freedom and *P* value noted<br>*Give P values as exact values whenever suitable.*
☒	☐	For Bayesian analysis, information on the choice of priors and Markov chain Monte Carlo settings
☒	☐	For hierarchical and complex designs, identification of the appropriate level for tests and full reporting of outcomes
☒	☐	Estimates of effect sizes (e.g. Cohen's *d*, Pearson's *r*), indicating how they were calculated

Our web collection on statistics for biologists contains articles on many of the points above.

Software and code

Policy information about availability of computer code

Data collection	Fluorescence microscope (Olympus BX51, CoolLED pE-4000) with Matlab 2019a and HCImageLive 4.4.1.0 were used to collect data for the microscope readout of this study. Matlab code can be made available on request.<br>Portable fluorescent reader (Axxin AX-2X-S) with Kinetic Designer Software V2.0 were used to collect data for the portable readout of this study.
Data analysis	Data analysis was performed in Matlab 2019a and R2022a, and described in methods. Matlab code can be made available on request.<br>The fitting software is open source and available at GitHub (https://github.com/bensmiller/detection-limit-fitting/).<br>GraphPad Prism V10.3.0 was used to generate plots and kinetic curves.

For manuscripts utilizing custom algorithms or software that are central to the research but not yet described in published literature, software must be made available to editors and reviewers. We strongly encourage code deposition in a community repository (e.g. GitHub). See the Nature Portfolio guidelines for submitting code & software for further information.

Data

Policy information about availability of data

All manuscripts must include a data availability statement. This statement should provide the following information, where applicable:

- Accession codes, unique identifiers, or web links for publicly available datasets
- A description of any restrictions on data availability
- For clinical datasets or third party data, please ensure that the statement adheres to our policy

All data generated or analysed during this study are available within the Article, its Supplementary Information file, and the Source data file. Source data are provided with this paper. The full image dataset and the computer code used are available from the corresponding author on request, in line with UCL and funder's requirements (UKHSA and EPSRC policy framework on research data).

Research involving human participants, their data, or biological material

Policy information about studies with human participants or human data. See also policy information about sex, gender (identity/presentation), and sexual orientation and race, ethnicity and racism.

Reporting on sex and gender	Our study didn't involve human participant or human data. Sex and gender were not considered in the study design.
Reporting on race, ethnicity, or other socially relevant groupings	Our study didn't involve human participant or human data. Race, ethnicity, or other socially relevant groupings were not considered in the study design.
Population characteristics	Our study didn't involve human participants.
Recruitment	No participant was recruited for this study.
Ethics oversight	Our study didn't need approval from an organisation.

Note that full information on the approval of the study protocol must also be provided in the manuscript.

Field-specific reporting

Please select the one below that is the best fit for your research. If you are not sure, read the appropriate sections before making your selection.

☒ Life sciences ☐ Behavioural & social sciences ☐ Ecological, evolutionary & environmental sciences

For a reference copy of the document with all sections, see nature.com/documents/nr-reporting-summary-flat.pdf

Life sciences study design

All studies must disclose on these points even when the disclosure is negative.

Sample size	As for the part of proof-of-concept study, determining analytical sensitivities, as opposed to clinical sensitivities, small sample sizes are suitable and no sample size calculation was necessary. 62 raw wastewater samples from Welsh National WBE programme were evaluated in this study. Details of the number of replicates for each figure are given in the text and figure captions.
Data exclusions	No data were excluded from analysis.
Replication	Reproducibility was ensured by measurements from multiple biological replicates. The exact number of independent biological experiments is mentioned in the figure legends. No results are included that were not observed in multiple experiments. All attempts at replication were successful.
Randomization	There was no randomisation as the assay development reply on the model samples from serial dilutions of target (synthetic RNA), so there were no covariates. The wastewater evaluation were designed to compare the diagnostic performance of the conventional pathogen identification. All samples were equally applied to both methods.
Blinding	Samples were randomised upon giving them to the study staff, who were kept blinded to the SARS-CoV-2 RT-PCR results of the samples when they performed validation experiments for the assay developed in this study.

Reporting for specific materials, systems and methods

We require information from authors about some types of materials, experimental systems and methods used in many studies. Here, indicate whether each material, system or method listed is relevant to your study. If you are not sure if a list item applies to your research, read the appropriate section before selecting a response.

Materials & experimental systems

n/a	Involved in the study	
☐	☒	Antibodies
☒	☐	Eukaryotic cell lines
☒	☐	Palaeontology and archaeology
☒	☐	Animals and other organisms
☒	☐	Clinical data
☒	☐	Dual use research of concern
☒	☐	Plants

Methods

n/a	Involved in the study	
☒	☐	ChIP-seq
☒	☐	Flow cytometry
☒	☐	MRI-based neuroimaging

Antibodies

Antibodies used	1. Anti-Digoxigenin antibody, Abcam, catalogue number: ab76907, goat polyclonal, lot number: GR3197960-3.<br>2. Antibodies used in the lateral-flow strips include polyclonal (rabbit) anti-FITC antibody and polyclonal (goat) digoxigenin antibody labelled with carbon black nanoparticles (for PCRD FLEX dipsticks strips). The antibodies were included as parts of the lateral-flow assay kit by Abingdon Health.
Validation	1. From manufacturer website: "ab76907 reacts 100% with digoxigenin and digoxin, it does not cross-react with the carrier protein KLH or other steroids."<br>Tested applications: IHC-P, WB, ELISA<br>Purified from immunized serum by ion exchange chromatography and immunoaffinity chromatography.<br>2. Validation was performed by the supplier (Abingdon Health).

Plants

Seed stocks	No seed stock or plant material was used.
Novel plant genotypes	No novel plant genotype was produced.
Authentication	No seed stock or plant material was used and no novel plant genotype was produced.
